# A Feedback Regulatory Loop Involving dTrbd/dTak1 in Controlling IMD Signaling in *Drosophila Melanogaster*

**DOI:** 10.3389/fimmu.2022.932268

**Published:** 2022-07-14

**Authors:** Yongzhi Hua, Yangyang Zhu, Yixuan Hu, Fanrui Kong, Renjie Duan, Chao Zhang, Chuchu Zhang, Shikun Zhang, Yiheng Jin, Yizhu Ye, Qingshuang Cai, Shanming Ji

**Affiliations:** ^1^ Centre for Developmental Biology, School of Life Sciences, Anhui Agricultural University, Hefei, China; ^2^ School of Preclinical Medicine, Wannan Medical College, Wuhu, China

**Keywords:** dTrbd/dTak1 association, dTrbd condensation and phase separation, dub enzymatical activity, IMD innate immune pathway, *Drosophila melanogaster*

## Abstract

Negative regulators of the inflammatory responses are essential for the maintenance of immune homeostasis and organismal fitness. In *Drosophila*, the deubiquitinase (Dub) dTrbd selectively restricts the K63-linked ubiquitination modification of dTak1, a pivotal kinase of the IMD signaling pathway, to regulate the IMD innate immune response. However, which domain and how it functions to enable dTrbd’s activity remain unexplored. Here, we provide compelling evidence showing that the NZF domain of dTrbd is essential for its association with dTak1. Meanwhile, the Linker region of dTrbd is involved in modulating its condensation, a functional state representing the Dub enzymatical activity of dTrbd. Of interest, the activated IMD signals following bacterial stimuli enhance the dTrbd/dTak1 interaction, as well as the condensate assembly and Dub enzymatical activity of dTrbd. Collectively, our studies shed light on the dual mechanisms by which the IMD signaling-mediated feedback loop of dTrbd/dTak1 precisely regulates the innate immune response in *Drosophila*.

## Introduction

As a rapid and critical defensive manner, innate immunity represents the first line of the host immune system (1–3). It is triggered by the pattern recognition receptors (PRRs), which are able to sense both extra-cellular germs and intra-cellular infections or damages within hours (1, 4). This triggering further initiates a non-specific inflammatory response, produces pleiotropic effective molecules without immune memory, and amplifies the protective reaction to other cells and organs to dominate adaptive immune responses (5).

*Drosophila melanogaster* has been one of the most well-known animal models for deciphering the fundamental molecular mechanisms underlying the precise regulation of host inflammatory reaction (2, 6). It harbors three main advantages: 1) as an insect whose defensive reaction only relies on innate immunity, *Drosophila* is an ideal model for investigating innate immune response without sophisticated interfering or cross-talk of adaptive immunity; 2) the great evolutionary congruence between *Drosophila* and mammals makes the pioneering discoveries regarding innate immunity of fruit flies be a potentially pivotal theoretic basis for studying mammalian systems (7); 3) performing genetic studies on *Drosophila* is highly convenient thanks to the powerful genetic manipulation tools and availability of various *Drosophila* mutants and transgenes.

In *Drosophila*, the innate immune response is mainly controlled by two signaling pathways, the Toll and the immune deficiency (IMD) pathways (2, 8, 9), which share substantial similarities with the mammalian MyD88-dependent Toll-like receptor (TLR) and the tumor necrosis factor receptor (TNFR) pathways, respectively (8, 10). The PRRs of both pathways sense the environmental pathogen-associated molecular patterns (PAMPs) and further activate a series of downstream caspase reactions, leading to the translocation of the transcription factor nuclear factor-kappa B (NF-κB) into the nucleus and the expression of a set of anti-microbial peptides (AMPs) (2, 6, 11, 12). Undoubtedly, a timely and robust innate immune response is pivotal for flies surviving in the battles against pathogenic invaders. Excessive activation of the innate immune response, however, is also deleterious, leading to detrimental consequences in various tissues/organs and even organismal mortality (8, 13–17). Thus, turning down the activated innate immune signaling pathways by negative regulators is fundamental for the maintenance of immune homeostasis and fitness (6, 8, 9, 16). It has been suggested that the IMD signaling pathway is precisely regulated by multiple negative modulators (8, 16). For instance, several proteins belonging to the peptidoglycan recognition protein (PGRP) family have been shown to be involved in the degradation of DAP- and Lys-type peptidoglycans (18–20), thereby antagonizing IMD signaling. Inside the cells, passive players such as Caspar (21), Cyld (22), Dnr1 (23, 24), dTrbd (13), Pirk (25, 26), Scny (27), and SkpA (28) have been demonstrated to play essential roles in down-regulating the IMD pathway through various regulatory manners. Among them, we noticed a deubiquitinase (Dub) dTrbd, which selectively modulates the K63-linked ubiquitination of dTak1 to attenuate IMD signaling. However, which domain is required for its biological function remains poorly understood.

In this study, we report a feedback regulatory loop involving the dTak1/dTrbd axis to control the IMD signaling pathway in *Drosophila*. We show that the N-terminal NZF domain of dTrbd is both required and sufficient for its binding to dTak1 for deubiquitination, whereas the Linker region mediates dTrbd condensation, which represents a functional state for executing its Dub enzymatical activity. Moreover, we find that bacterial stimuli-induced IMD activation not only reinforces the physical association of dTrbd/dTak1, but also positively facilitates dTrbd condensation to enhance the Dub enzymatical activity of dTrbd, thus negatively contributing to IMD signaling. Collectively, our results uncover dual mechanisms involving dTrbd/dTak1 to regulate the IMD signaling-mediated inflammatory reaction in *Drosophila*.

## Results

### Minor Alterations in dTrbd Expression During IMD Signaling

To explore whether dTrbd expression tightly parallels IMD signaling, we challenged *Drosophila* S2 cells with heat-killed Gram-negative bacterium *Escherichia coli* (*E. coli*) as previously described (29) and examined the mRNA profiles of *dTrbd* at various time points (0, 6, and 12 hours) *via* reverse transcription plus quantitative polymerase chain reaction (RT-qPCR) assays. As illustrated in **Figure S1A**, *dTrbd* transcript levels remained relatively similar along with the activation of the IMD signals, which were monitored by looking at the mRNA levels of the downstream AMPs genes *attacin A* (*AttA*) and *cecropin A1* (*CecA1*) (**Figures S1B, C**) (30). Consistent results were obtained when we examined the protein levels of endogenous dTrbd in S2 cells by Western blot assays (**Figures S1D, E**) utilizing newly-generated anti-dTrbd antibodies (see Materials and Methods). To further validate our findings *in vivo*, we challenged male *w^1118^* adults with freshly cultured *E. coli*. At various time points post bacterial infection (0, 6, and 12 hours), we collected fat body tissues from these flies to examine the dynamic expression profiles of *dTrbd*. As shown in **Figures S1F-H**, *dTrbd* was barely altered at the mRNA and protein levels in response to bacterial infection. Collectively, these results imply that *dTrbd* is not induced by pathogenic challenging both in cultured S2 cells and in *Drosophila*. Of note, the mRNA occurrences of *dTak1*, whose translational products are the regulatory targets of dTrbd in controlling the IMD signals (13), also displayed relatively potty amplitude fluctuations during IMD signaling both *in vitro* and *in vivo* (**Figures S1I, J**). These results were consistent with the previous findings in a microarray assay (25).

### dTrbd Physically Associates With dTak1 *via* the N-terminal NZF Domain

**Figure 1:**
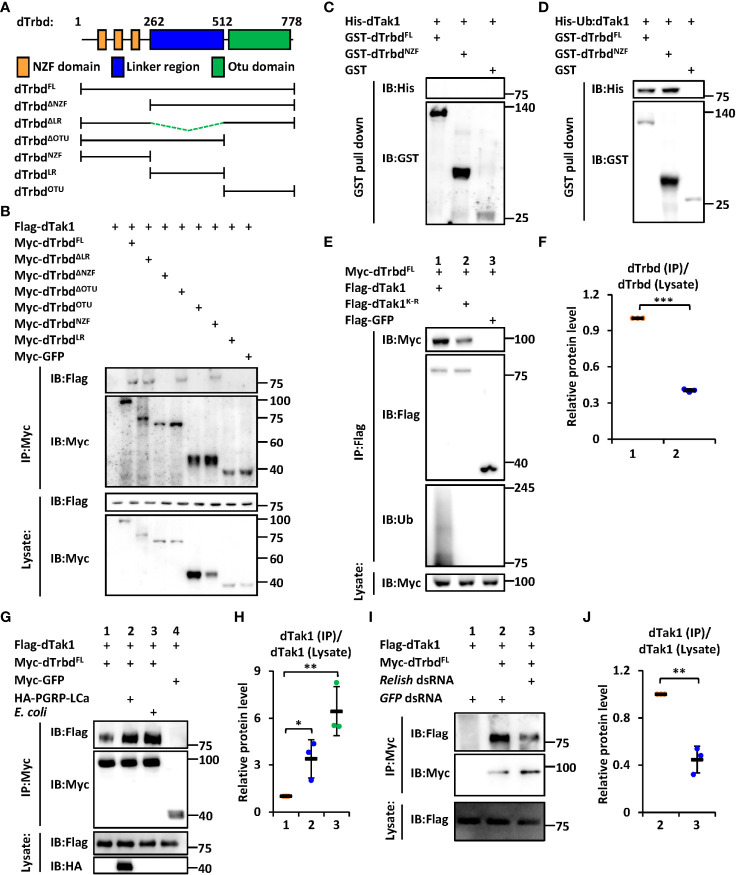
dTrbd associates with dTak1 *via* the N-terminal NZF domain. **(A)** Domain architecture of dTrbd. **(B)** Various combinations of expressing plasmids were transfected into S2 cells as indicated. 48 h post transfection, cells were lysed for immunoprecipitation assays using anti-Myc agarose beads, followed by Western blot assays. **(C, D)** Purified His-tagged dTak1 **(C)** or Ub-dTak1 **(D)** were incubated with GST-tagged dTrbd^FL^ or dTrbd^NZF^ (GST was used as the control). Samples were then subjected to GST pull-down and Western blot assays. **(E, F)** Indicated expressing plasmids were transfected into S2 cells for 48 h. Cell lysates were then prepared for immunoprecipitation and Western blot assays. Densitometry analysis to quantify the protein levels of Myc-dTrbd in the co-immunoprecipitant **(E)** is shown in **(F)**. **(G, H)** S2 cells were transfected with various combinations of expressing plasmids for 36 h, followed by treatment of heat-killed *E coli* (Lane 4 in G) or PBS (Lanes 1-3 in G) for 12 h. Cells were then harvested for immunoprecipitation and Western blot assays as indicated **(G)**. Densitometry analysis to quantify the protein levels of Flag-dTak1 in the co-immunoprecipitant **(G)** is shown in **(H) (I, J)** S2 cells were treated with different dsRNAs for 48 h and transfected with various expressing plasmids for 48 h as indicated. Cell lysates were then prepared for immunoprecipitation and Western blot assays **(I)**. Densitometry analysis to quantify the protein levels of Flag-dTak1 in the co-immunoprecipitant **(I)** is shown in **(J)**. In **(F, H, J)** data are shown as mean ± SD. *p < 0.05; **p < 0.01; ***p < 0.001.

We next sought to investigate the functional domain of dTrbd essential for its interaction with dTak1. The region between the N-terminal Npl4 Zinc-Finger (NZF) domain and the C-terminal Ovarian Tumor (OTU) domain was referred to as the Linker region (LR, **Figure 1A**). We constructed various truncated forms of dTrbd expressing plasmids (**Figure 1A**), namely dTrbd^FL^, dTrbd^NZF^, dTrbd^LR^, dTrbd^OTU^, dTrbd^ΔNZF^, dTrbd^ΔLR^, and dTrbd^ΔOTU^, and transfected them together with plasmids expressing dTak1 into *Drosophila* S2 cells for co-immunoprecipitation (co-IP) experiments (GFP expressing or empty plasmids were utilized as the controls). As illustrated in **Figure 1B**, the N-terminal conserved NZF domain is both required and sufficient for the physical association between dTrbd and dTak1.

It has been suggested that the NZF domain functions mainly as a type of Ubiquitin-binding domains (31, 32). Based upon previous findings that dTak1 can undergo ubiquitination to regulate IMD signaling (13), we raised two explanations for the NZF domain binding to dTak1: 1) the NZF domain interacts with Ubiquitin, which behaves (directly or indirectly) as a “connective bridge” for the dTrbd/dTak1 association; 2) the NZF domain is also able to bind to other proteins/residues (dTak1 in our case) besides Ubiquitin. To further prove these hypotheses, we purified GST-tagged dTrbd^FL^ and dTrbd^NZF^, as well as His-tagged dTak1 proteins from *E. coli* (**Figure S2A**), where it was not ubiquitinated (**Figure S2B**). As shown in the GST pull-down assays, we did not observe any detectable interactions of purified His-dTak1 with either GST-tagged dTrbd^FL^ or dTrbd^NZF^ (**Figure 1C**), suggesting dTrbd physically associates with dTak1 largely through Ubiquitin or indirectly. We next constructed a plasmid expressing dTak1 with a Ubiquitin at its N-terminus (referred to as Ub-dTak1) (**Figure S2C**). This fusion protein was further proven to be easily bound by both dTrbd^FL^ and dTrbd^NZF^ in the GST pull-down assays (**Figure 1D**), suggesting that the NZF domain of dTrbd is able to associate directly with the Ubiquitin-conjugated dTak1. To obtain more evidence, we selectively mutated all the lysine residues of dTak1 into arginines (referred to as dTak1^K-R^). Further co-IP experiments in *Drosophila* S2 cells displayed that dTak1^K-R^ still readily co-immunoprecipitated with dTrbd when ubiquitination modifications were nearly fully abolished (**Figures 1E, F**). Notably, mutation of all the lysine residues in dTak1 somehow weakened its binding affinity to dTrbd (**Figures 1E, F**). Taken together, our results imply that dTrbd not only binds to the ubiquitinated dTak1, but also interacts with the non-ubiquitinated dTak1 through a(some) potential intermediator(s).

### dTrbd Associates With dTak1 in an IMD Signal-Dependent Manner

To explore the involvement of IMD signaling in the binding of dTrbd to dTak1, we treated S2 cells with heat-killed *E. coli* or transfected them with plasmids expressing the constitutively active form of PGRP-LCa (33) to activate the IMD signaling pathway. These treatments indeed markedly induced downstream AMP expressions in S2 cells (**Figures S3A, B**). Our further co-IP approaches showed that the physical association between dTrbd and dTak1 was greatly strengthened when the IMD pathway was activated in S2 cells (**Figures 1G, H**). This observation was consistent with our *in vivo* examination of the dTrbd/dTak1 association (**Figures S4A, B**). In addition, silencing *relish* by the treatment of *relish* dsRNA in S2 cells largely prevented the interaction between dTrbd and dTak1 (**Figures 1I, J**), demonstrating that the IMD signaling pathway plays a critical role in impacting the dTrbd/dTak1 association.

### The LR Regulates the Dub Enzymatical Activity of dTrbd

**Figure 2:**
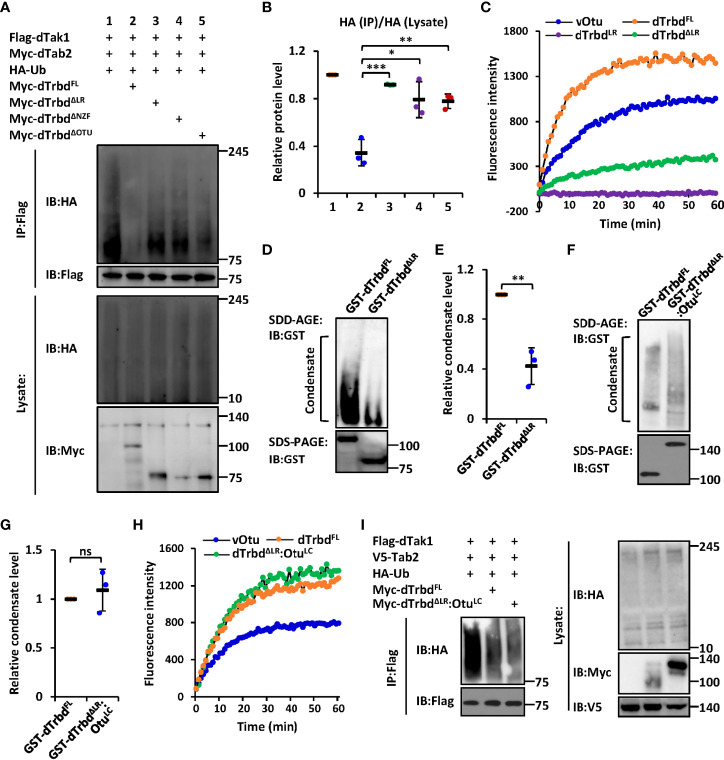
The LR-mediated condensation of dTrbd is essential for its Dub enzymatical activity. **(A, B)** S2 cells were transfected with various expressing plasmids for 48 h, followed by ubiquitination assays to examine the ubiquitination patterns of dTak1 as indicated **(A)**. Densitometry analysis to quantify the ubiquitination levels of dTak1 **(A)** is shown in **(B)**. **(C)** *In vitro* Dub assays displayed the enzymatical activity of purified proteins including dTrbd^FL^, dTrbd^LR^, and dTrbd^ΔLR^. The viral Otu (vOtu) was used as the control. **(D–G)** Purified proteins [GST-tagged dTrbd^FL^ and dTrbd^ΔLR^ in **(D)** as well as GST-tagged dTrbd^FL^ and dTrbd^ΔLR :^ Otu^LC^ in **(F)**] were subjected to SDD-AGE and SDS-PAGE assays. Densitometry analyses to quantify the condensate levels of indicated proteins **(D, F)** were shown in (E, G). **(H)** *In vitro* Dub assays showed that purified dTrbd^ΔLR :^ Otu^LC^ enabled to cleave Ubiquitin from the substrate in the reaction. **(I)** Various combinations of expressing plasmids were transfected into S2 cells for 48 h, followed by ubiquitination assays as indicated. In **(B, E, G)** data are shown as mean ± SD. *p < 0.05; **p < 0.01; ***p < 0.001; ns, not significant.

We next sought to identify the functional requirements of the domain(s) for dTrbd executing its Dub enzymatical activity. We transfected dTak1 expressing plasmids together with various truncated forms of dTrbd expressing plasmids into S2 cells and performed ubiquitination assays. As shown in **Figures 2A, B** and **S5A**, deletion of either the NZF or the OTU domain markedly prevented the functional roles of dTrbd in restricting the ubiquitination levels of dTak1 in S2 cells. It is not surprising for the authors to observe these results since the OTU domain is a necessarily catalytical center for dTrbd (13, 34), and the NZF domain is essential for dTrbd binding with its target (dTak1 in our case, **Figure 1**) for the catalytical reaction of deubiquitination.

The observation that over-expression of dTrbd^ΔLR^ largely lost the negative impact on the ubiquitination level of dTak1 in S2 cells (**Figures 2A, B** and **S5A**) prompted us to explore how the LR is involved in modulating the enzymatical ability of dTrbd. To do this, we first performed an *in vitro* Dub assay (35) using purified proteins namely dTrbd^FL^, dTrbd^LR^, and dTrbd^ΔLR^ to quantify the requirements of the LR for dTrbd’s Dub enzymatical activity (in this assay, the Ub-Rhodamine110 was used as the reaction substrate). As illustrated in **Figure 2C**, loss of the LR markedly down-regulated but not fully abolished the Dub enzymatical activity of dTrbd. Of note, dTrbd^LR^ reasonably displayed no apparent Dub activity, since it is absent of the catalytical triad of the OTU domain (**Figure 2C**).

We then performed semi-denaturing detergent agarose gel electrophoresis (SDD-AGE) assays to examine the condensation levels of purified dTrbd^FL^ and other truncated forms of dTrbd proteins, as condensate assembly has recently been suggested to play essential roles in regulating protein enzymatical activities (33, 36–39). As shown in **Figures 2D, E**, signals representing aggregated forms of tested proteins could be easily detected in dTrbd^FL^ but not in dTrbd^ΔLR^, suggesting that the LR is required for dTrbd to form condensates. Of note, the LR itself is readily condensed *in vitro* (**Figure S6A**). The above observations are rather similar to our previous findings that the low-complexity (LC) domain-mediated condensation is essential for the Dub enzymatical activity of *Drosophila* Otu (33). However, the bioinformatic analysis suggested very low disordered property of dTrbd^LR^ (**Figure S6B**). To our surprise, when we further replaced the LR of dTrbd with the LC domain of Otu, we found that the purified dTrbd^ΔLR :^ Otu^LC^ protein easily formed condensate in our SDD-AGE assays (**Figures 2F, G**) and displayed comparable Dub enzymatical activity (**Figure 2H**), implying that dTrbd condensation is critical for its Dub enzymatical activity. Consistent results were also obtained when we further monitored the condensation patterns and Dub enzymatical activities of dTrbd^FL^ and dTrbd^ΔLR :^ Otu^LC^ in S2 cell cultures (**Figures 2I**, **S6C**).

### dTrbd Undergoes Liquid-Liquid Phase Separation

**Figure 3:**
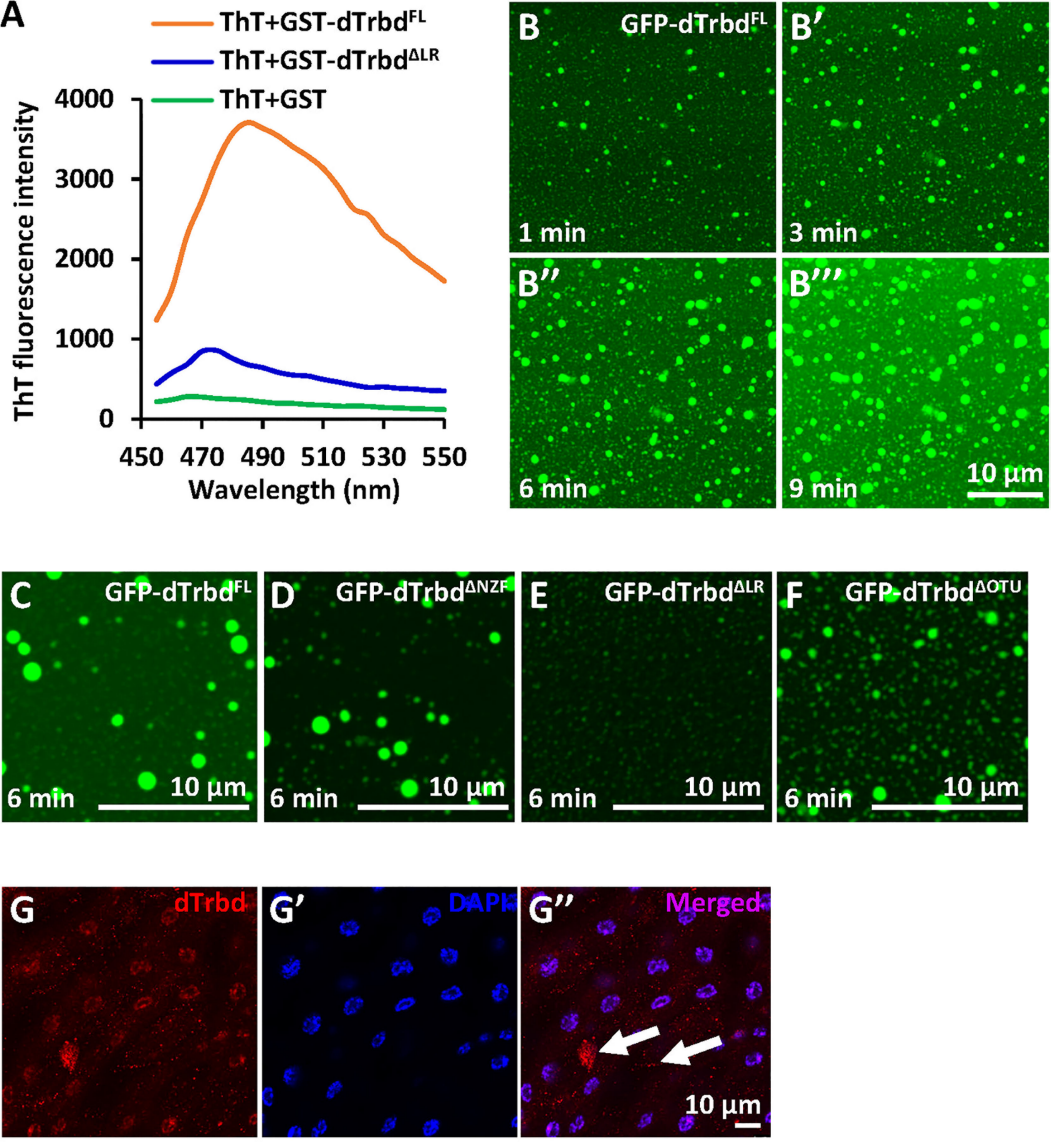
dTrbd undergoes phase separation depending on the Linker region. **(A)** dTrbd^FL^ but not dTrbd^ΔLR^ binds to the amyloid binding dye Thioflavin T (ThT). (**B-B’’’**) Phase separation of dTrbd^FL^ at various time points (1, 3, 6, and 9 min, respectively). Scale bars, 10 μm. **(C-F)** Different truncated forms of dTrbd (dTrbd^FL^, dTrbd^ΔNZF^, and dTrbd^ΔOtu^) can undergo phase separation except dTrbd^ΔLR^. Scale bars, 10 μm. **(G)** Confocal imaging of *w^1118^* larval fat bodies that were stained with anti-dTrbd (red) antibody. Scale bars, 10 μm.

According to our knowledge that a large amount of protein condensates can undergo phase separation to form membraneless organelles or granules, thus modulating enzymatical properties of proteins and the related biological processes (33, 36–39), we thus sought to identify whether the dTrbd condensates behave in a similar fashion. We first subjected purified dTrbd^FL^ and dTrbd^ΔLR^ to ThT binding assays (33) to examine whether dTrbd could form amyloidal fibers *in vitro*. As shown in **Figure 3A**, dTrbd^FL^ but not dTrbd^ΔLR^ easily bound to ThT. We then fused dTrbd^FL^ and various truncated forms of dTrbd with GFP at their N-terminuses. Under confocal microscope, we observed that GFP-dTrbd^FL^ underwent phase separation (**Figures 3B-B’’’**). In addition, both dTrbd^ΔNZF^ and dTrbd^ΔOTU^ also easily formed dense structures in a fairly short time except for dTrbd^ΔLR^ (**Figures 3C-F**). We last examined whether dTrbd forms dense structure *in vivo*. As illustrated in the immunostaining assays, dTrbd condensates can be easily observed in the fat body cells (**Figures 3G-G’’**).

**Figure 4:**
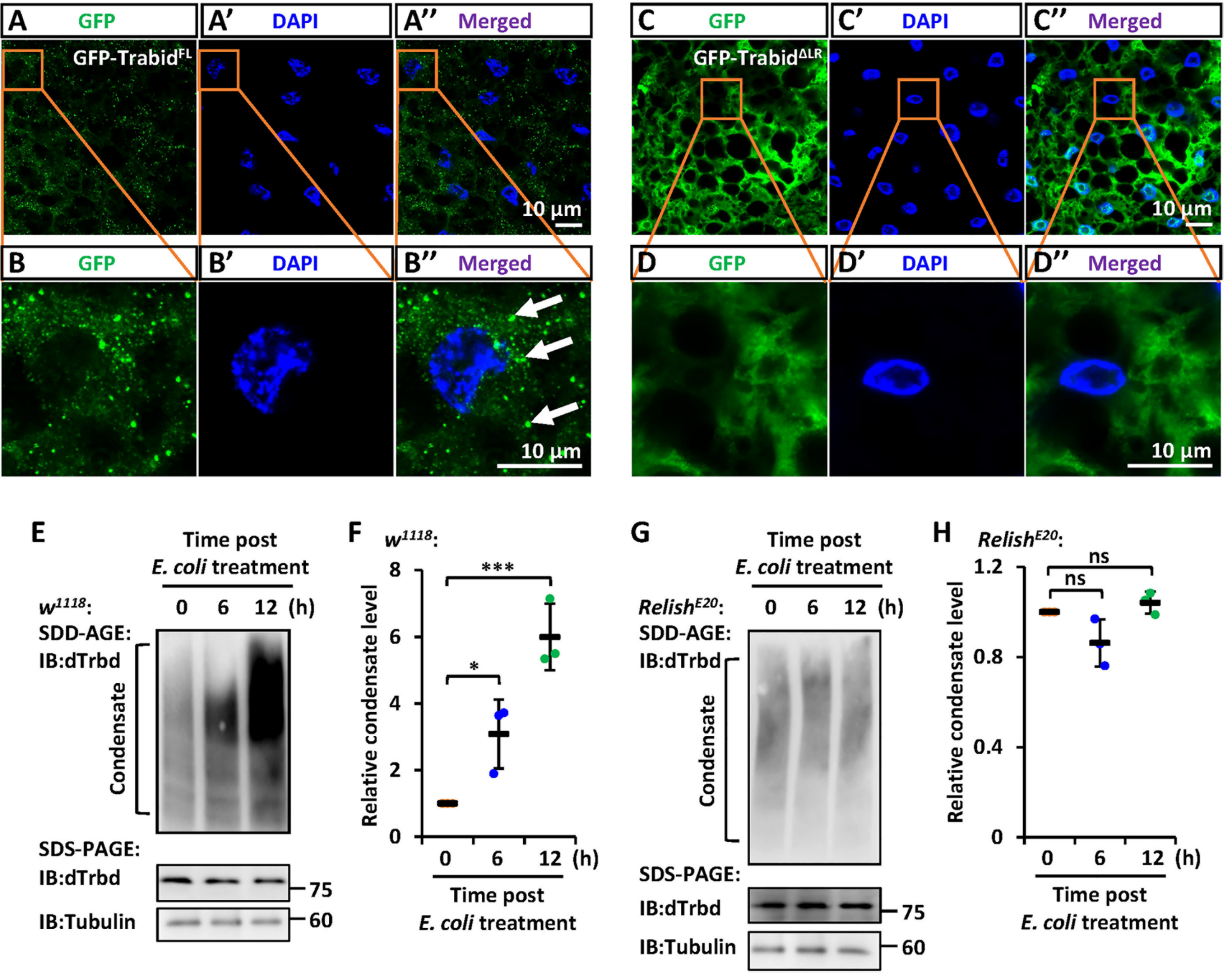
IMD signaling regulates the LR-mediated condensation of dTrbd. **(A–D)** Confocal imaging of fat bodies dissected from larvae including *c564^ts^>GFP-dTrbd^FL^* **(A, B)** and *c564^ts^>GFP-dTrbd^ΔLR^* **(C, D)**. Scale bars, 10 μm. **(E, F)** Male *w^1118^* adults were infected with freshly-cultured *E. coli*. At various time points (0, 6, and 12 h) post infection, fat bodies were dissected and subjected to SDD-AGE (upper panel in **E**) or SDS-PAGE (lower panel in **E**) assays. Densitometry analysis to quantify the condensate level of samples in **(E)** is shown in **(F)**. **(G, H)** Similar as in **(E, F)** except the *Relish^E20^* mutants were used. In **(F, H)** data are shown as mean ± SD. *p < 0.05; ***p < 0.001; ns, not significant.

To further explore the involvement of the LR in the condensate assembly of dTrbd *in vivo*, we constructed two types of transgenic flies including P{*Uasp-GFP-dTrbd^FL^*} and P{*Uasp-GFP-dTrbd^ΔLR^*}, and crossed them with flies of P{*c564-gal4*}, which is a fat body specific driver (40). In the fat body cells of these progenies, we found that GFP-dTrbd^FL^ other than GFP-dTrbd^ΔLR^ formed dense structures (**Figures 4A-D’’**).

### Dynamic Regulation of the dTrbd Condensate During IMD Signaling

We next sought to explore whether IMD signaling affects the condensate assembly of dTrbd. We infected male *w^1118^* flies with freshly-cultured *E. coli*, and dissected the fat bodies at various time points. As shown in the SDD-AGE assays, bacterial challenging dramatically increased the dTrbd condensation (**Figures 4E, F**). These results imply that activation of the IMD signaling pathway positively contributes to dTrbd condensation, thereby enhancing its Dub enzymatical activity to turn down the IMD signaling. Consistently, in the *Relish* loss-of-function mutants (*Relish^E20^*), we failed to observe apparent alterations of dTrbd condensate (**Figures 4G, H**).

### dTrbd Functions *In Vivo via* Multiple Domains

**Figure 5:**
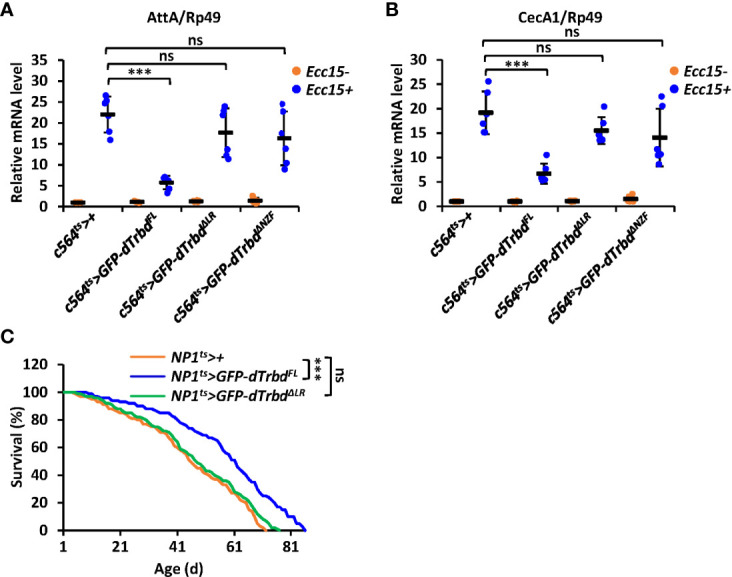
dTrbd functions *in vivo via* multiple domains. **(A, B)** Male adults including *c564^ts^>+* (control), *c564^ts^>GFP-dTrbd^FL^*, *c564^ts^>GFP-dTrbd^ΔNZF^*, *c564^ts^>GFP-dTrbd^ΔLR^*, and *c564^ts^>GFP-dTrbd^ΔLR^* were infected with freshly cultured *Ecc15* (*Ecc15* +) or not (*Ecc15* -). 12 h later, flies were harvested and lysed for RT-qPCR assays to examine the mRNA levels of *AttA* **(A)** or *CecA* **(B)**. **(C)** Male adults including *NP1^ts^>+* (control), *NP1^ts^>GFP-dTrbd^FL^*, and *NP1^ts^>GFP-dTrbd^ΔLR^* were subjected to lifespan assays. In **(A, B)** data are shown as mean ± SD. ***p < 0.001; ns, not significant.

To further confirm the requirements of both the NZF domain and the LR for dTrbd functioning *in vivo*, we injected *Erwinia carototovovora Asp 15* (*Ecc15*) into adult flies including 1) *c564^ts^>+* (control), 2) *c564^ts^>GFP-dTrbd^FL^*, 3) *c564^ts^>GFP-dTrbd^ΔNZF^*, and 4) *c564^ts^>GFP-dTrbd^ΔLR^*. We observed much lower mRNA occurrences of *AttA* and *CecA1* in dTrbd^FL^ overexpressing (*c564^ts^>GFP-dTrbd^FL^*) flies compared to those of the control (**Figures 5A, B**). However, the mRNA levels of these AMP genes were relatively comparable between samples from *c564^ts^>GFP-dTrbd^ΔNZF^*, *c564^ts^>GFP-dTrbd^ΔLR^*, and the control flies (**Figures 5A, B**), suggesting both the NZF domain and the LR are required for dTrbd to negatively govern IMD signaling post microbial infection.

Previous studies have shown that preventing the IMD signaling pathway in gut tissues positively contributes to the life longevity of *Drosophila* (15, 41–43). We thus sought to identify whether dTrbd modulates lifespan *via* multiple domains. As shown in **Figure 5C**, flies with ectopic expression of dTrbd^FL^ in the guts displayed a prolonged lifespan. Additionally, overexpression of dTrbd without the LR was dispensable for impacting the fly lifespan (**Figure 5C**), strengthening the conclusion that dTrbd relies on multiple structural domains to execute its functional role *in vivo*.

## Discussion

To date, various contributors have been thought to play passive roles in controlling the *Drosophila* IMD signaling pathway (44–46), providing potential theoretical bases for understanding how NF-κB dependent immune reactions are tightly regulated to avoid excessive inflammation. However, in the presence of so many negative modulators, how can IMD signaling be efficiently activated to clear invading microbes? One would naturally propose a possible working model in which the activities of these negative factors are precisely controlled during IMD signaling. In this study, we focus on a Dub dTrbd, which has recently been shown to restrict the K63-linked ubiquitination of dTak1, thereby attenuating IMD signaling in flies (13). We show that although dTrbd is not induced upon activation of the IMD signals by microbial stimuli, its binding ability to dTak1 and Dub enzymatical processivity are enhanced. Collectively, our studies uncover an IMD signaling-dependent feedback regulatory loop involving dTrbd/dTak1 to precisely control *Drosophila* innate immune response *via* diverse mechanisms.

As mentioned in the Introduction and above, the ubiquitinated dTak1 is targeted by the Dub dTrbd for deubiquitination to prevent Imd signaling (13). Nevertheless, which domain is essential for dTrbd to fulfill its biological role remains unexplored. To address this issue, we first explored whether dTrbd associates with dTak1 in a domain-dependent manner. In the domain mapping and the GST pulldown assays, we observed that dTrbd binds to dTak1 *via* its N-terminal conserved NZF domain. Interestingly, this domain has long been considered as a typical Ubiquitin binding domain (31, 32), which is consistent with our findings that dTrbd^NZF^ can only bind physically to purified Ub-dTak1 but not dTak1. However, in cultured *Drosophila* S2 cells, our point mutation and co-IP experiments imply that dTrbd can interact with non-ubiquitinated dTak1. Put together, these data prompted us to hypothesize that dTrbd is capable of binding not only to the ubiquitinated dTak1 but also to the non-ubiquitinated dTak1 *via* an(some) intermediator(s). This hypothesis is consistent with our findings that dTrbd/dTak1 association is mediated by IMD signaling. A highly possible regulator of the IMD pathway, for instance, is the *Drosophila* Tab2 (dTab2), which has been shown to associate with both dTrbd (13) and dTak1 (47, 48). To our knowledge, there has been no evidence supporting the notion that dTab2 is able to bind to Ubiquitin, although the mammalian TAB2 homologs have been proven to harbor a relatively high Ubiquitin-binding affinity *via* the NZF domain (49). It will be worthwhile to identify the potential dTab2/Ubiquitin association and its involvement in dTrbd interacting with dTak1.

Recently, protein condensation and phase separation have been shown to participate in regulating the mammalian innate immune responses *via* impacting various cytosolic signaling pathways, namely the retinoic acid-inducible gene I protein (RIG-I) (50), the cyclic GMP-AMP synthase (cGAS)-stimulator of interferon genes (STING) (51), and the NF-κB (52) pathways. Unfortunately, very few studies have focused on the relationships between phase separation and inflammation in insects. We and colleagues recently found that In *Drosophila*, protein condensation and phase separation can mediate the Dub enzymatical activity of Otu to negatively contribute to the IMD innate immune pathway *via* the low-complexity (LC) domain (33). In this study, we demonstrate that the Dub enzymatical activity of dTrbd is also affected by the assembly of the functional condensate. However, dTrbd condensation is surprisingly dependent on the Linker region lacking LC properties, implying diverse regulatory mechanisms by which protein condensation mediates the Dub enzymatical processivity and the related biological processes. Notably, an outstanding study by Kleino *et al.* suggested that the RHIM motif in proteins such as Imd, PGRP-LC, and PGRP-LE is required for the assembly of functional amyloids (53). Therefore, it is thus probable that such motif exists in dTrbd and plays a role in governing its behavior.

## Materials and methods

### *Drosophila* Strain and Husbandry

All flies used in this study were raised in standard *Drosophila* culture medium (corn flour and agar) and cultured at 25°C. The *w^1118^* strain was used as the control and the host for P-element-mediated transformation. The following Gal4 lines and transgenes were used in this study: 1) P{*c564-Gal4*}, P{*NP1-Gal4*}, and P{*Tub-gal80^ts^*} were obtained from Bloomington stock center; 2) P{*UAS-GFP-dTrbd^FL^*}, P{*UAS-GFP-dTrbd^ΔNZF^*}, and P{*UAS-GFP-dTrbd^ΔLR^*}, in which the full-length dTrbd, dTrbd^ΔNZF^, and dTrbd^ΔLR^ were placed under the control of the UASp promoter, respectively.

### Antibodies

The following primary antibodies were used for Western blot and immunostaining assays: Mouse anti-Flag (1:5000, Sigma-Aldrich, Cat#F1804); Rabbit anti-Myc (1:3000, Medical & Biological Laboratories, Cat#562); Mouse anti-dTrbd (1:1000), which was generated to an N-terminal fragment of dTrbd (amino acids 155-253) fused to His and purified from *E. coli*; Rabbit anti-dTak1 (1:1000, Abcam, Cat#239353); Mouse anti-β-Tubulin (1:3000, Cowin, Cat#CW0098M); Rabbit anti-HA (1:2500, Medical & Biological Laboratories, Cat#561); Mouse anti-GFP (1:2000, Sigma-Aldrich, Cat#G6539); Mouse anti-V5 (1:5000, Sigma-Aldrich, Cat#V8012).

The secondary antibodies for Western blot and immunostaining assays include Goat anti-Mouse IgG H&L (HRP); Goat anti-Rabbit IgG H&L (HRP); Goat anti-Mouse IgG H&L (Alexa Fluor 555) (1:1000, Abcam, Cat#ab150078).

### RT-qPCR Assays

Total RNA was isolated with Trizol Reagent (Invitrogen), followed by cDNA synthesis using the first-strand cDNA synthesis kit (Transgen) according to the manufacturer’s instructions. RT-qPCR was performed in triplicate using SYBR Green Master Mix (Thermo) on a Light Cycler 480. Concentrations of specific targets were normalized to endogenous reference *Rp49*. Data shown are relative mRNA abundance compared to that of the control. Primers used in RT-qPCR assays were shown in **Supplementary Table 1**.

### S2 Cell Transfection and Co-IP Assays

S2 cells were cultured in insect medium (Gibco) supplemented with 10% fetal bovine serum (FBS, Hyclone) at 27°C. Lipofectamine 2000 (Invitrogen) was used for transfection of all cells in this study. For Co-IP assays, cells were lysed in lysis buffer (150 mM NaCl, 50 mM Tris-HCl, pH 7.5, 10% glycerol, 0.5% Triton X-100, 10 mg/ml aprotinin, 10 mg/ml leupeptin, and 1 mM phenylmethylsulfonyl fluoride). Anti-Myc agarose beads (Abmart) or Anti-Flag agarose beads (Sigma) were used for indicated immunoprecipitation experiments. Immunoprecipitants were subjected to Western blot assays to detect the indicated protein levels.

### Protein Purification and GST Pull-Down Assays

The GST-tagged or His-tagged proteins were expressed and purified from *E. coli* strain BL21. Briefly, protein expression was induced by adding isopropyl-beta-D-thiogalactopyranoside (final concentration of 1 mM) into BL21 bacterial medium when the OD_600_ of the culture reached 0.4-0.6, followed by overnight incubation at 18°C. Bacterium were pelleted and resuspended in 15 ml of lysis buffer (25 mM Tris-HCl, pH 7.5, 100 mM NaCl, 2 mM EDTA). Cell integrity was disrupted by sonicating each sample for 30 min using an EpiShear™ Probe Sonicator (pulse 4 s on, 6 s off, 40% amplitude). Protein content was collected as the supernatant of the following centrifugation at 10,000 rpm, 4°C for 20 min. The Glutathione Sepharose 4B (Sigma, 17-0756-01) or BeyoGold™ His-tag Purification Resin (Beyotime, P2218) were used for purification of GST-tagged or His-tagged proteins, respectively. Each sample (1 μg) was finally resuspended in loading buffer and subjected to the Coomassie brilliant blue assay.

For GST pull-down assay, indicated proteins were incubated with the Glutathione Sepharose 4B (10 μl for each sample) at 4°C for 3 h. Samples were then washed with wash buffer (PBS with 1% TritonX-100) 3 times (1 h in total) and subjected to Western blot assays.

### RNAi in S2 Cells by Treatment of dsRNA

All dsRNAs were synthesized using the *in vitro* T7 transcription Kit (Promega) according to the manufacture’s protocol. S2 cells were harvested and then diluted into fresh medium at a density of 1×10^6^ cells per ml and treated with dsRNAs immediately. 1 h later, FBS (Hyclone) was added to the culture medium at a final concentration of 10%. Primers for dsRNA synthesis are shown in **Supplementary Table 1**.

### SDD-AGE Assays

For samples of cell lysate, transfected S2 cells were harvested and lysed with lysis buffer (50 mM Tris-HCl, pH 7.5, 150 mM NaCl, 0.5% Triton X-100, 10% glycerol, and 1mM phenylmethylsulfonyl fluoride) over ice for 30 min, followed by centrifugation (13000 rpm at 4°C) for 10 min. Supernatant was carefully collected and loaded with loading buffer (0.5 × TBE, 10% glycerol, 2% SDS, 0.0025% bromophenol blue) at room temperature for 15 min. For samples of purified proteins, indicated amounts of proteins were directly loaded with loading buffer at room temperature. Newly prepared 1.5% agarose gel with 0.1% SDS was pre-run by electrophoresis in running buffer (1×TBE and 0.1% SDS) for 1 h at 4°C. Loaded protein samples were then subjected to electrophoresis for 1 h and transferred to PVDF membrane (Millipore) for immunoblotting analysis.

### *In Vitro* DUB Assays

The Ub rhodamine 110 (Boston Biochem) was used as the substrate for *in vitro* deubiquitination assays. In brief, proteins (100 ng for each sample) with Ub rhodamine 110 were incubated in the assay buffer (150 mM NaCl, 5 mM MgCl_2_, 50 mM Tris-HCl, pH 7.5). The mixture was added into 384-well low volume plate and dynamic fluorescence was monitored with excitation and emission wavelengths set at 485/20 and 535/20 nm, respectively, utilizing the MD SpectraMax M5 Microplate Reader. Fluorescence intensity for each condition was plotted as a function of time.

### Ubiquitination Assays

Transfected S2 cells were harvested and lysed in lysis buffer (50 mM Tris-HCl, pH 7.5, 150 mM NaCl, 0.5% Nonidet P-40, 10% glycerol, and 1% SDS). Samples were then heated at 95°C for 5 min and combined with binding buffer (50 mM Tris-HCl, pH 7.5, 150 mM NaCl, 0.5% Nonidet P-40, and 10% glycerol) to adjust the SDS to a final concentration of 0.1%. Lysates were subjected to sonication and immunoprecipitated utilizing anti-Myc agarose beads (Abmart) or anti-Flag M2 beads (Sigma) for 4 h. The immune complexes were washed with wash buffer (50 mM Tris-HCl, pH 7.5, 500 mM NaCl, 0.5% Nonidet P-40, and 10% glycerol) 3 times (1 h in total), and subjected to Western blot assays to detect the ubiquitination patterns of indicated proteins.

### Phase Separation

Purified proteins (10 mg/ml) were incubated at room temperature with 50 mM Tris-HCl, pH 7.5, and 150 mM NaCl. Protein phase separation was examined using Zeiss LSM 710 Meta confocal microscope, and images were captured at indicated time points.

### Immunostaining Assays

Fat bodies were dissected from larvae and fixed in fix buffer (4% formaldehyde, 0.25% Tween-20, and 75% n-heptane in 1×PBS) for 30 min at room temperature. Samples were then washed 3 times (total 1 h) and blocked (2 h) in PBTA buffer (0.3% Tween-20 and 1.5% BSA in 1×PBS). After incubation with Mouse anti-dTrbd (diluted in PBTA) antibody at 4°C overnight, samples were washed 3 times (total 1 h) at room temperature, and then incubated with Goat anti-Mouse (Alexa Fluor 555) antibody (diluted in PBTA) at room temperature for 2.5 h with DAPI (1:1000) to mark the cell nucleus. Finally, samples were washed 3 times (1 h in total), pressed into tablets, and observed under confocal microscope.

### Pathogenic Infection of Flies and Larvae

Overnight bacterial culture was collected and diluted in sterile 1×PBS dilution at a concentration of OD_600 =_ 1. Male adult flies were injected with 4.6 nl of the diluted bacterium or the same volume of PBS as controls. For larvae, they were washed three times with sterile H_2_O and placed in a small drop of water on a black rubber block. Injection was performed using a sharp capillary in the posterolateral body with 100 nl bacterial suspension (same volume of PBS was used for the control). Treated flies or larvae were transferred to a vial containing regular food and collected for further analysis as indicated.

### Statistical Analyses

All statistical analyses were performed by using GraphPad Prism 8. Data were shown as mean and standard errors. Statistical significance was determined by using the two-tailed Student’s *t* or Mann-Whitney tests except for lifespan assays, in which the Log-Rank test (Kaplan-Meier method) was used for statistical analyses. The p value of less than 0.05 was considered statistically significant. * p < 0.05; ** p < 0.01; *** p < 0.001; ns, not significant.

## Data Availability Statement

The raw data supporting the conclusions of this article will be made available by the authors, without undue reservation.

## Author Contributions

YHua, QC, and SJ conceived and designed the experiments. YHua, YHu, FK, RD, CZ, CCZ, SZ, YJ, YY, and QC performed the experiments. YHua, YZ, YHu, QC, and SJ analyzed the data. YHua, YZ, and YHu performed statistical analyses. YHua, QC, and SJ wrote the manuscript. All authors contributed to the article and approved the submitted version.

## Funding

This work was supported by grants from the National Natural Science Foundation of China (31871470 and 32100702) and the Anhui Provincial Natural Science Foundation (2008085J14).

## Conflict of Interest

The authors declare that the research was conducted in the absence of any commercial or financial relationships that could be construed as a potential conflict of interest.

## Publisher’s Note

All claims expressed in this article are solely those of the authors and do not necessarily represent those of their affiliated organizations, or those of the publisher, the editors and the reviewers. Any product that may be evaluated in this article, or claim that may be made by its manufacturer, is not guaranteed or endorsed by the publisher.
