# Corrigendum: A feedback regulatory loop involving dTrbd/dTak1 in controlling IMD signaling in *Drosophila Melanogaster*

**DOI:** 10.3389/fimmu.2022.993987

**Published:** 2022-08-03

**Authors:** Yongzhi Hua, Yangyang Zhu, Yixuan Hu, Fanrui Kong, Renjie Duan, Chao Zhang, Chuchu Zhang, Shikun Zhang, Yiheng Jin, Yizhu Ye, Qingshuang Cai, Shanming Ji

**Affiliations:** ^1^ Centre for Developmental Biology, School of Life Sciences, Anhui Agricultural University, Hefei, China; ^2^ School of Preclinical Medicine, Wannan Medical College, Wuhu, China

**Keywords:** dTrbd/dTak1 association, dTrbd condensation and phase separation, dub enzymatical activity, IMD innate immune pathway, *Drosophila melanogaster*

Qingshuang Cai was not included as corresponding author in the published article. The Corresponding Author details appear below:

Qingshuang Cai, 17610679696@163.com; Shanming Ji, jism@ahau.edu.cn

The authors apologize for this error and state that this does not change the scientific conclusions of the article in any way. The original article has been updated.

## Publisher’s note

All claims expressed in this article are solely those of the authors and do not necessarily represent those of their affiliated organizations, or those of the publisher, the editors and the reviewers. Any product that may be evaluated in this article, or claim that may be made by its manufacturer, is not guaranteed or endorsed by the publisher.

