# Supplementary material for: A Feedback Regulatory Loop Involving dTrbd/dTak1 in Controlling IMD Signaling in Drosophila Melanogaster
Source: Front Immunol. 2022 Jul 14;13:932268. doi: 10.3389/fimmu.2022.932268 (PMC9329959; doi:10.3389/fimmu.2022.932268)
Supplement: Supplementary file 1 [file DataSheet_1.pdf]

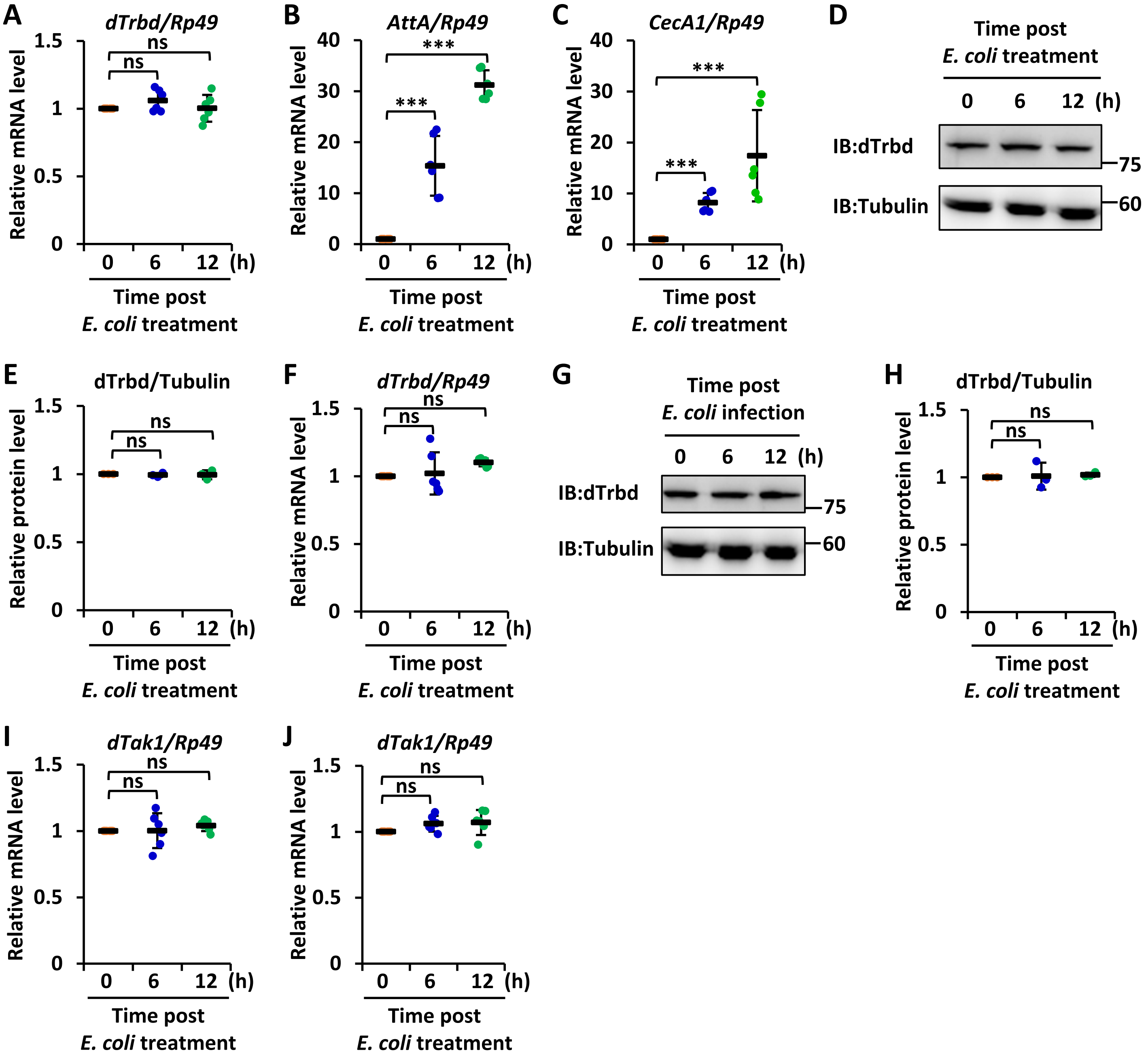

Supplementary Figure 1. Minor alterations of dTrbd/dTak1 expression during IMD signaling. **(A-E)** *Drosophila* S2 cells were treated with heat-killed *E. coli*. At indicated time points (0, 6, and 12 h) post treatment, cells were lysed for RT-qPCR assays to monitor the mRNA levels of *dTrbd* (A), *AttA* (B), and *CecA1* (C), or for Western blot assays (D) to quantify the protein level of dTrbd. Densitometry analysis to quantify the expression levels of dTrbd in (D) is shown in (E). Tubulin was used as the loading control. **(F-H)** Male w^{1118} adults were infected with freshly cultured *E. coli*. At various time points (0, 6, and 12 h), fat bodies were dissected, followed by RT-qPCR (F) or Western blot (G) assays to examine the mRNA or protein levels of dTrbd. Densitometry analysis to quantify the expression levels of dTrbd in (G) is shown in (H). Tubulin was used as the loading control. **(I, J)** S2 cells or male w^{1118} adults were challenged with heat-killed (I) or freshly cultured (J) *E. coli* as indicated. At various time points (0, 6, and 12 h), samples (cells or dissected fat bodies) were subjected to RT-qPCR assays. In A-C, E, F, and H-J, data are shown as mean $\pm$ SD. * $p < 0.05$, ** $p < 0.01$; *** $p < 0.001$; ns, not significant.

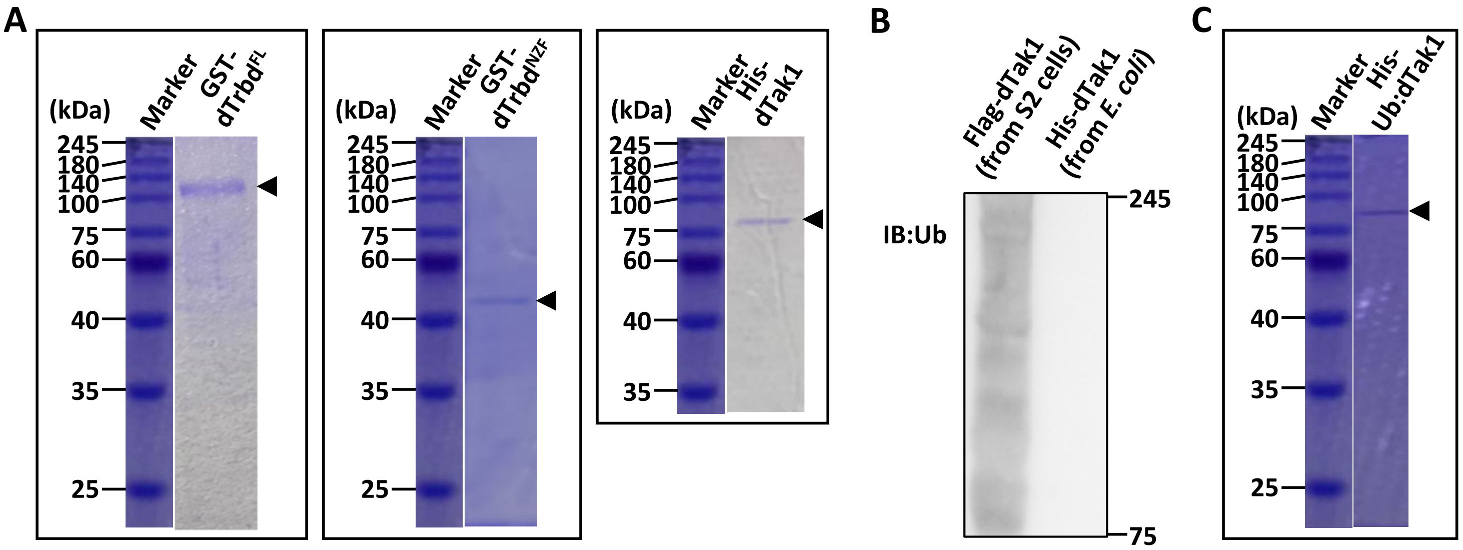

Supplementary Figure 2. dTrbd associates with dTak1 via the N-terminal NZF domain.
(A) Coomassie brilliant blue staining of purified GST-dTrbd^{FL}, GST-dTrbd^{NZF}, and His-dTak1. For each sample, 1 µg indicated protein was loaded. **(B)** Purified Flag-dTak1 and His-dTak1 (100 ng for each sample) were loaded and subjected to Western blot assay using anti-Ub antibody. **(C)** Coomassie brilliant blue staining of purified His-Ub:dTak1 (1 µg).

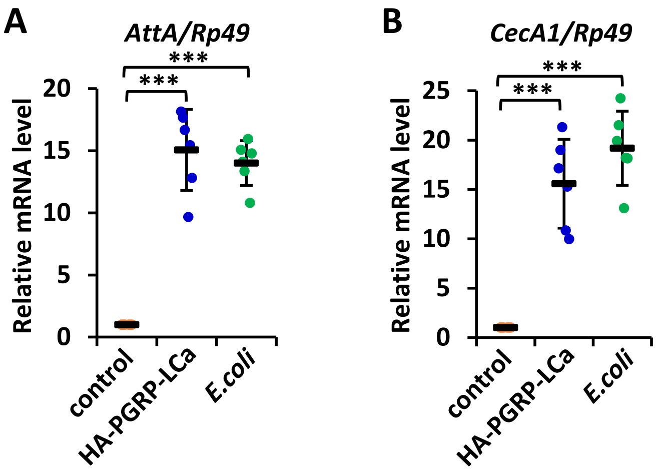

Supplementary Figure 3. Activation of IMD signaling by over-expression of PGRP-LCa or treatment of heat-killed *E. coli* in S2 cells.

(A, B) S2 cells were transfected with indicated expressing plasmid or treated with heat-killed *E. coli* for 12 h. Cells were then lysed for RT-qPCR assays to monitor the mRNA levels of *AttA* (A) or *CecA1* (B). Data are shown as mean ± SD. *** $p < 0.001$.

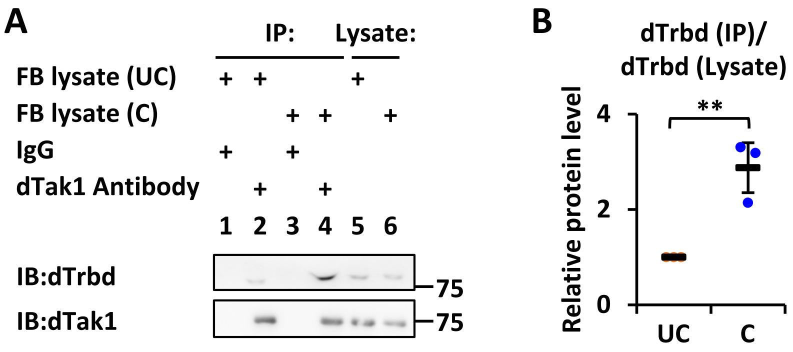

Supplementary Figure 4. dTrbd and dTak1 form a complex *in vivo*.
(A, B) Larvae of w^{1118} were infected with *E. coli* (challenged, C) or sterile PBS (unchallenged, UC) for 12 h. Fat bodies were dissected and lysed for immunoprecipitation using anti-dTak1 antibody (IgG was used as control). Immunoprecipitants were washed and subjected to Western blot assays using indicated antibodies (A). Densitometry analysis to quantify the levels of indicated proteins is shown in (B). Data are shown as mean ± SD. ** $p < 0.01$.

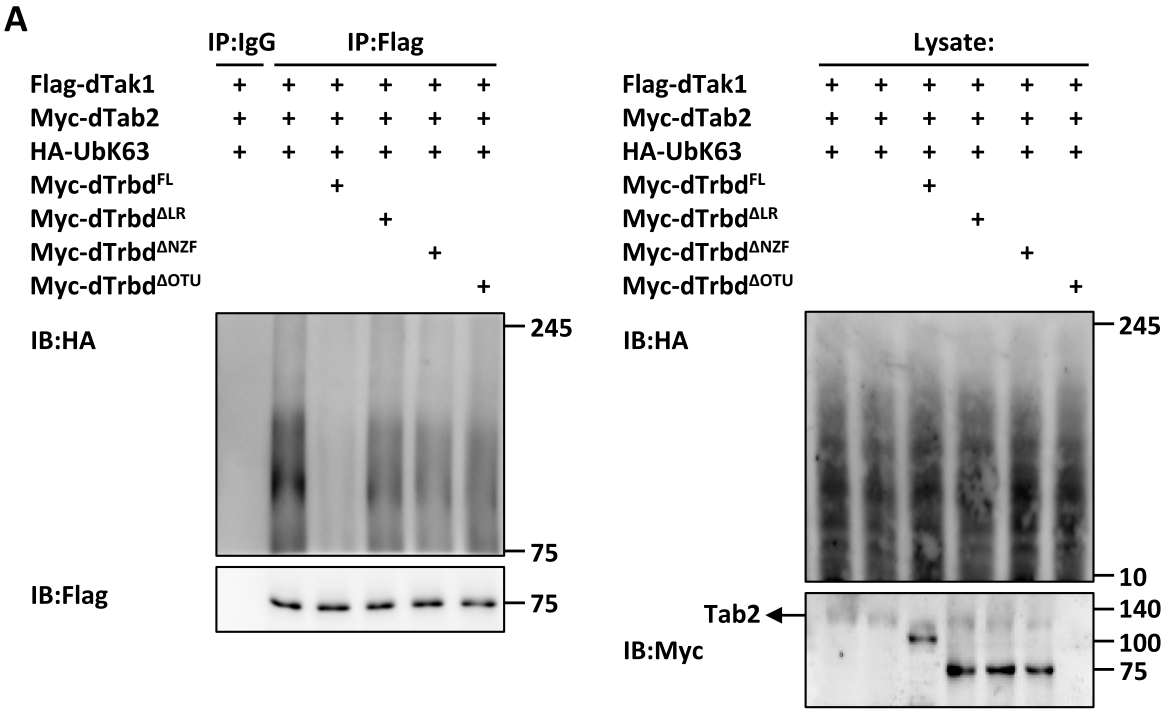

Supplementary Figure 5. dTrbd restricts the K63-linked ubiquitination of dTak1 via multiple domains. **(A)** S2 cells were transfected with indicated expressing plasmid for 48 h. Cells were then lysed for ubiquitination assays to monitor the K63-linked ubiquitination of dTak1.

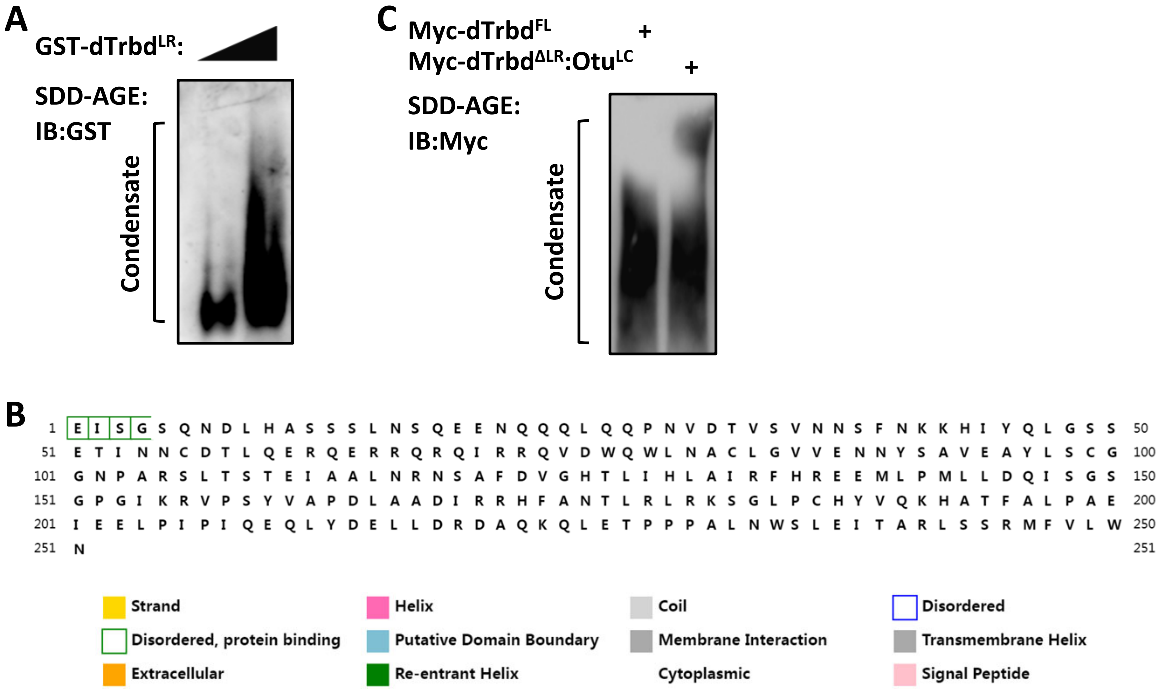

Supplementary Figure 6. The LR of dTrbd is essential for its condensation.
(A) Different doses (50 ng and 200 ng) of purified GST-dTrbd^{LR} were loaded and subjected to SDD-AGE assay. **(B)** Bioinformatical analysis of the LR of dTrbd (http://bioinf.cs.ucl.ac.uk/psipred) to determine the low complexity property. **(C)** S2 cells were transfected with expressing plasmids (1 μg for each plasmid) for 48 h as indicated. Cells were then lysed and subjected for SDD-AGE assays.
